# Mental Health and Substance Use Disorders in Transplant Waitlist, VAD, and Heart Transplant Patients: A TriNetX Database Analysis

**DOI:** 10.3390/jcm13113151

**Published:** 2024-05-28

**Authors:** Chloe Grzyb, Dongping Du, Balakrishnan Mahesh, Nandini Nair

**Affiliations:** 1 Heart and Vascular Institute, Pennsylvania State University College of Medicine, Hershey, PA 17033, USA; cgrzyb@pennstatehealth.psu.edu; 2 Department of Industrial, Manufacturing, and Systems Engineering, Texas Tech University, Lubbock, TX 79409, USA; dongping.du@ttu.edu; 3 Division of Cardiothoracic Surgery, Department of Surgery, Penn State Health Milton S. Hershey Medical Center, Hershey, PA 17033, USA; bmahesh@pennstatehealth.psu.edu; 4 Division of Cardiology, Department of Medicine, Penn State Health Milton S. Hershey Medical Center, Hershey, PA 17033, USA

**Keywords:** substance use, cardiac transplant, VAD, mental health

## Abstract

**Background/Objectives**: Mental health and substance use disorders (MHDs and SUDs) affect cardiac allograft and VAD recipients and impact their quality of life and compliance. Limited research currently exists on MHDs and SUDs in this population. **Methods**: This study compares the incidence of MHDs and SUDs in the transplant list, VAD, and post-transplant patients with that in heart failure patients. Study cohorts were derived from the TriNetX database using ICD-10 codes. Differences in incidence were examined using the log-rank test. Adults with MHDs and SUDs before the window of time were excluded. All comparisons were made between propensity-matched cohorts. Statistical significance was set at *p* < 0.05. **Results**: Transplant waitlist patients showed a significant increase in the incidence of anxiety, depression, panic, adjustment, mood, alcohol use, and eating disorders. Post-transplant patients showed a significant increase in depression and opioid use. VAD patients showed a significant increase in depression and a decrease in panic disorder and anxiety. These results allow for further investigations on prevention and coping strategies. **Conclusions**: The deterioration of mental health can significantly impact medication compliance, survival, and quality of life. Opioid use for pain management in the early postoperative period should be further investigated to assess its impact on long-term substance use and addiction.

## 1. Introduction

The global prevalence of mental health disorders has been on the rise and affects the quality of life in heart failure patients [1,2]. Depression and anxiety have been noted to affect more than 30% of the transplant population, which can influence outcomes in this population [3]. Patients with advanced heart failure may wait years on the cardiac transplant list before undergoing heart transplantation. Others may be candidates for ventricular assist devices (VADs) as a bridge to transplant or as a destination therapy for life. The uncertainty, fear, and medical challenges of these experiences may have a significant impact on patients’ mental health. Depression affects solid-organ recipients, and most studies suggest depression is an independent risk factor for increased morbidity and mortality after transplant [1,2,3,4,5]. Pre-transplant depression can influence outcomes such as medication compliance and hospitalizations but did not show a significant impact on post-transplant survival [2,4]. Poor mental health has been shown to decrease treatment compliance and increase hospitalizations/infectious complications in post-transplant patients [4]. The use of immunosuppressive drugs, especially corticosteroids and calcineurin inhibitors, as well as continued medical issues and health anxiety, likely contribute to deterioration in mental and physical health [2]. It has been shown that physical activity improves frailty [6]. Motivation to be physically active and exercise is influenced by emotional well-being and therefore significantly impacts the quality of life [6,7].

After cardiac transplantation or VAD implantation, concerns such as diet, adjustment, lifestyle, medication regimen, and follow-up care are closely monitored, especially in the months following surgery. However, psychiatric disorders may present during the preoperative and anytime in the postoperative course, thus requiring lifelong attention. This emphasizes the need for multidisciplinary care coordination and support for heart failure patients while waiting, before, and after surgical intervention. Rehabilitation should address physical well-being and cognitive and psychological functioning, which are needed to extend beyond the postoperative period.

Elevated rates of depression, anxiety, and decreased QOL have been identified in post-transplant patients and inconsistently in VAD patients [8,9,10,11]. However, there is limited evidence as to the effect of other mental health disorders (MHDs) and SUDs. This study compares the incidence of a range of MHDs and SUDs in the transplant waitlist, VAD, and post-transplant patients with that of heart failure patients to help pave the way for further investigations in this area, which will impact the quality of life and outcomes in these populations.

## 2. Materials and Methods

TriNetX is a global health research network that compiles decoded data from the electronic health records of many healthcare organizations. Hence, research does not require an institutional review board review [12]. TriNetX includes large academic medical institutions across the world. The data are stored on servers or through a virtual platform at participating institutions that are subsequently collected and aggregated. Demographic, diagnostic, and clinical data were used. Decoded data were used from the Global Collaborative Network consisting of 104 healthcare organizations in 30 countries. The database includes insured and uninsured patients. This study is Health Insurance Portability and Accountability Act (HIPAA)-compliant and is exempt from IRB approval. The process by which datasets are deidentified by TriNetX is attested through a formal determination by a qualified expert, as defined in Section §164.514(b) (1) of the HIPAA Privacy Rule. Protected Health Information (PHI) is therefore made available to the users of the platform in a deidentified format.

The analysis process included two main steps: (1) defining the cohorts through query criteria and (2) setting up and running the analysis. Setting up the analysis required definitions for the index event, outcomes criteria, and the time frame. Outcomes were compared using four analyses: measures of association, survival, number of instances, and lab result distribution. These analyses have additional options, including the outcomes’ definitions and analysis specifications. Propensity score matching was used in this study. The first three analyses support the setting designated as “exclude patients with outcomes before the window”. The measure of association analysis calculates and compares the fraction of patients with the selected outcome.

Survival analysis was performed using the Kaplan–Meier test to estimate the probability of the outcome at a respective time interval (the daily time interval was used in this analysis). To account for the patients who exited the cohort during the analysis period, censoring was applied. In this analysis, patients were removed from the analysis (censored) after the last fact in their record.

In the analysis involving the number of instances, we calculated how many times the outcome occurred in the time window. This analysis included two additional settings: “include patients with zero instances” and “the definition of an instance”. Selecting to exclude patients with zero instances would remove these patients from the calculations for mean number of instances, standard deviation, or median.

Table 1 shows the ICD codes used in this study for diagnosis. The incidence of MHDs and SUDs was compared in adult patients with heart failure with those who were on the transplant waitlist, had VADs, and those who received cardiac transplant within 15 years of the index event. Index events that occurred up to 20 years ago were included. An index event was defined as the time the patient entered the cohort. This was set to the first diagnosis of heart failure for control patients and the date of transplant/VAD insertion or first registration for those on the cardiac transplant waitlist. Differences in incidence were examined using the log-rank test and hazard ratios. Patients with mental health disorders before the time window were excluded. Patients between ages 0 and 100 were included. Propensity score matching was performed using TriNetX (https://ctsi.psu.edu/research-support/trinetx, Transplant and transplant waitlist on 28 September 2023, and Vad list on 29 September 2023) to balance demographic and medical comorbidities. Statistical significance was set at *p* ≤ 0.05.

## 3. Results

### 3.1. Transplant Waitlist Patients

After propensity score matching, 29,900 patients were identified in each cohort. The mean ages at the index event were 49.3 +/− 21.5 for heart failure patients and 64.6 +/− 18.7 for transplant waitlist patients. The characteristics of this cohort are shown in Table 2.

Transplant waitlist patients showed a statistically significant increase in the incidence of anxiety, panic disorder, adjustment disorder, depression, alcohol use disorder, and eating disorder. Survival analysis showed a statistically significant impact of anxiety, panic disorder, adjustment disorder, depression, mood disorder, nicotine dependence, opioid use, dementia, and eating disorders on the waitlist patients. The other tested parameters were not significant, as shown in Table 3.

### 3.2. Cardiac Transplant Patients

After propensity score matching, 4855 patients were identified in each cohort. The only variable that differed significantly between the two groups was age, which was higher in heart failure patients, as shown in Table 4. The mean age at index was 68.9 +/− 16.7 for heart failure patients and 44.8 +/− 20.9 for transplant patients.

Post-transplant patients showed a statistically significant 1.5–2-fold increase in the incidence of adjustment disorder, depression, and opioid use compared to the heart failure population, as noted in Table 5. Survival analysis showed a statistically significant impact of a heart transplant on adjustment, mood, and opioid use disorders. The other tested parameters were not significant.

### 3.3. VAD Patients

In total, 10,659 patients were identified in each cohort after propensity score matching. The VAD cohort was older than the heart failure cohort (61.3 +/− 16.4 vs. 34.1 +/− 20.3 years, respectively). The other baseline characteristics shown in Table 6 were comparable between groups.

VAD patients showed a statistically significant increase in the incidence of depression and a statistically significant decrease in panic disorder and anxiety. No significant difference was noted in the incidence of somatoform, adjustment, mood, alcohol, eating, opioid disorders, PTSD, nicotine use, or self-harm. Survival analysis (13.6 years) showed a statistically significant impact of anxiety, panic disorder, PTSD, adjustment, depression, eating disorder, self-harm, opioid/alcohol use, and nicotine dependence, as noted in Table 7. Interestingly, opioid use in this population seemed to confer a survival benefit with a hazard ratio of 0.74. A marginal but significant survival benefit was also noted with nicotine dependence. Both of these factors showed a nonsignificant decrease in incidence in the VAD population.

## 4. Discussion

The majority of studies about mental health and transplant/VAD patients to date focus on depressive and anxiety symptoms in cardiac transplant patients. We attempted to perform a comprehensive analysis of MHDs and SUDs in three subgroups of heart failure patients using the largest patient cohort size to date.

### 4.1. Transplant List Patients

The waitlist period can last days to years. Absolute contraindications are a history of medical noncompliance and poor social support [13]. Some studies suggest that psychiatric conditions may negatively affect transplant outcomes through poor adherence, self-injurious behaviors, drug interactions, and poor social support on the whole [13].

Our results revealed that cardiac transplant waitlist patients had a statistically significant increase in the incidence of six psychiatric disorders (anxiety, depression, panic, adjustment, alcohol use, and eating disorders), whereas in the post-transplant patients, significant increases in incidence were noted only in adjustment disorder and depression. In the VAD population, a significant increase in incidence was only noted in depression, while anxiety and panic disorder were significantly decreased.

The trends noted in this study may be because the waitlist period is critical in maintaining eligibility for transplants and a time of uncertainty, fear, and stress. Early diagnosis, treatment, and improved access to resources for patients on the transplant waitlist are critical in improving survival and candidacy for transplant patients. This also highlights inequity between those who may have better social support and increased access to care and resources, which need further investigation.

The literature regarding posttransplant outcomes of patients with psychiatric disorders is inconsistent [14]. Studies cite a history of suicide/self-harm, depressive episodes, and poor medical adherence as the greatest factors impacting post-transplant survival time [14,15,16]. However, a comprehensive review contradicted these findings, suggesting that there is no clear association between prior psychiatric illness and morbidity and mortality and that patients with psychiatric conditions can have good outcomes after transplant [14,15,16,17]. Candidacy for transplant should be made through consideration of individual risk factors, not the presence of psychiatric conditions, to avoid stigmatization of this patient population.

### 4.2. Cardiac Transplant Patients

Our results revealed that transplant patients had a significant increase in the incidence of depression and opioid use disorder compared to heart failure patients. This supports existing research suggesting that transplant increases the risk of depression, often due to difficulty in coping with a lifestyle change and complications [4,18,19]. Interestingly, it is believed that the rates of anxiety and depression are lower in patients receiving heart transplants compared to those undergoing other cardiac surgeries, including valve replacement [20].

Studies have found that cardiac transplant patients are at increased risk (3–10%) of developing opioid use disorder. Severe pain during the recovery period may increase the risk of long-term dependence on opioids [19]. Decreasing the dose and duration of opioids prescribed at discharge may decrease the risk of long-term opioid use and dependence.

### 4.3. VAD Patients

Our results revealed that VAD patients had a statistically increased incidence of depression and a statistically significant decrease in panic disorder and anxiety following VAD implantation. Some evidence shows an initial improvement in depression and anxiety after implantation; however, patient-reported outcomes remained lower than those of transplant patients [9,21,22]. Our study looked at the longitudinal development of these disorders on a scale of over ten years, whereas these studies looked at weeks–month-long periods. Further investigation into the timing and onset of anxiety and depression is warranted.

VADs increase the risk of PTSD or panic disorder, despite the possibility of acute VAD dysfunction or mechanical failure [9]. Similarly, our results found a statistically significant decrease in the incidence of PTSD and anxiety following VAD insertion compared to heart failure patients. Existing research is primarily focused on anxiety, depression, and PTSD only.

Studies have found that depression and anxiety increase prior to VAD implantation, decline again after surgery, and resurface with complications and difficulty with adjustment [9,10]. While services are aimed at all phases, counseling and psychiatric services are generally concentrated around surgical intervention [8,23,24]. Years after the transplant, the patient may still struggle to cope and have recurrent medical complications. Patients with depression have been found to have elevated rates of stroke and sepsis [3]. Resources are most concentrated around the pre-and postoperative period. MHDs and SUDs are known to impact treatment compliance as well as compliance with lifestyle interventions, which is an important factor in predicting survival. Screening, counseling, and pharmacologic treatment should be offered when necessary.

This study was limited by its retrospective nature. Transplant candidates and VAD/transplant recipients may have more frequent interactions with the healthcare system and may be more likely to be diagnosed with an MHD or SUD than patients with heart failure. Some misidentification is possible with the use of a large, national database. Because the database was intended for billing purposes, it lacks granularity and may be missing data, which could contribute to underreporting of MHDs/SUDs. We were also unable to account for comorbid SUDs and MHDs. Further studies should assess the time to onset of mental health disorders and trends in correlation with treatment advances. There is also a need to determine the optimal management of patients with pre-existing MHDs in addition to those who develop disorders.

## 5. Conclusions

This is the first report on an extensive analysis of different MHDs and SUDs in the adult heart failure population undergoing advanced cardiac surgical therapies that impact a change in the quality of life postoperatively. We used propensity score matching to decrease intrinsic differences in confounding variables among the groups to ensure that data were more robust. These results lay the groundwork for further studies to investigate current prevention and strategies in place for transplant candidates as well as transplant and VAD patients. Increased rates of MHDs and SUDs are likely multifactorial. Early diagnosis, treatment, and improved access to resources for VAD, transplant candidates, and recipients may improve outcomes. The incorporation of longitudinal psychiatric care may improve patient quality of life and survival.

The limitations of this work are that it is a retrospective analysis that uses ICD codes with no interactions with any patient, which excludes any personal clinical insight.

Future investigations should include randomized prospective studies. Additionally, AI-driven algorithms should be used to study risk factors for MHDs and SUDs in this population on data collected prospectively. The identification of risk factors can help develop risk prediction models to improve patient selection and achieve better patient outcomes.

## Figures and Tables

**Table 1:** Diagnosis ICD codes used.

Diagnosis	ICD Codes
Heart failure (LV failure)	I50.1
VAD	Z95.811
Heart transplant list	Z94.1
Heart transplant recipient	33,945
Depression	F32, F33
Mood disorder	F39
Anxiety	F41.1
Panic disorder	F41.0
PTSD	F43.1
Adjustment disorder	F43.2
Eating disorders	F50
Somatoform disorders	F50
ADHD	F90
Opioid use disorders	F11
Alcohol use disorders	F10
Nicotine dependence	F17
Dementia	F03
Suicidal ideation or self-harm	R45.851, R45.88, T14.91, T50.902, X71-X83

**Table 2:** Propensity score matching for transplant waitlist versus heart failure patients.

Characteristic	Before Matching			After Matching		
	Heart Failure Patients	Transplant List Patients	Standard Difference	Heart Failure Patients	Transplant List Patients	Standard Difference
Age at index, years	49.3 +/− 21.5	69.1 +/− 16.5	1.029	49.4 +/− 21.5	64.6 +/− 18.7	0.755
Male	163,210 (57.6%)	20,259 (67.7%)	0.208	20,361 (68.1%)	20,217 (67.6%)	0.01
Race						
White	148,374 (52.4%)	17,628 (58.9%)	0.131	17,576 (58.8%)	17,607 (58.9%)	0.002
American Indian or Alaska Native	498 (0.2%)	95 (0.3%)	0.029	99 (0.3%)	95 (0.3%)	0.002
Pacific Islander	1273 (0.4%)	80 (0.3%)	0.31	90 (0.3%)	80 (0.35)	0.006
Black or African American	23,891 (8.4%)	5130 (17.1%)	0.263	5291 (17.7%)	5105 (17.1%)	0.016
Asian	7954 (2.8%)	658 (2.2%)	0.39	627 (2.1%)	658 (2.2%)	0.007
Essential hypertension	130,274 (46.0%)	10,664 (35.5%)	0.213	10,906 (36.5%)	10,664 (35.7%)	0.017
Neoplasms	61,898 (21.9%)	5867 (19.6%)	0.056	5808 (19.4%)	5860 (19.6%)	0.004
Diabetes mellitus	72,647 (25.7%)	7237 (24.2%)	0.034	7264 (24.3%)	7224 (24.2%)	0.003
Obesity and overweight	45,565 (16.1%)	4255 (14.2%)	0.052	4212 (14.1%)	4249 (14.2%)	0.004
Psychosocial stressors	13,436 (4.7%)	899 (3.0%)	0.09	856 (2.9%)	899 (3.05)	0.009
Chronic lower respiratory diseases	60,391 (21.3%)	4738 (15.8%)	0.142	4741 (15.9%)	4735 (15.8%)	0.001
Epilepsy or seizures	6007 (2.1%)	689 (2.3%)	0.012	661 (2.0%)	686 (2.3%)	0.017
Cerebral infarction	19,796 (7.0%)	1978 (6.6%)	0.015	1845 (6.2%)	1974 (6.6%)	0.018
Ischemic heart diseases	109,963 (38.8%)	9370 (31.3%)	0.159	9293 (31.1.%)	9368 (31.3%)	0.005
Heart failure	96,068 (33.9%)	13,166 (44.0%)	0.207	13,054 (43.7%)	13,120 (43.9%)	0.004
Atrial fibrillation and flutter	69,909 (24.7%)	6655 (22.2%)	0.058	6491 (21.7%)	6638 (22.2%)	0.012
Cardiomyopathy	41,677 (14.7%)	10,244 (34.2%)	0.466	10,140 (33.9%)	10,198 (34.1%)	0.004
Fibrosis and cirrhosis of the liver	5393 (1.9%)	1189 (4.0%)	0.123	1052 (3.5%)	1173 (3.9%)	0.021
Chronic kidney disease and acute kidney failure	74,403 (26.3%)	9736 (32.5%)	0.137	9711 (32.5%)	9693 (32.4%)	0.001
Number of patients	283,188	29,946		29,900	29,900	

**Table 3:** Incidence of MHDs/ SUDs in transplant waitlist patients versus heart failure patients.

	Incidence			Survival Analysis	
Disorder	Incidence in Cardiac Transplant Waitlist Patients	Incidence in Control (Heart Failure)	*p*-Value for Incidence Analysis	Hazard Ratio	Hazard Ratio *p*-Value
	Expressed as the Total Number in Propensity-Matched Cohort	Expressed as the Total Number in Propensity-Matched Cohort			
Somatoform disorder	261/29,773	144/29,759	0.86	1.57	0.4
Anxiety	1289/28,969	505/29,254	0.001 *	1.771	0.003 *
Panic disorder	552/29,460	260/29,557	0.042 *	1.486	0.032 *
PTSD	496/29,344	205/29,645	0.117	1.686	0.079
Adjustment disorder	1495/28,298	638/29,269	0.00 *	1.742	0.00 *
Depression	4293/25,149	2194/25,819	0.00 *	1.5	0.00 *
Mood disorder	600/29,587	281/29,598	0.126	1.463	0.004 *
Nicotine dependence	1459/27,603	1292/24,832	0.913	0.706	0.012 *
Alcohol use disorder	792/28,615	785/27,658	0.015 *	0.685	0.466
Opioid use	495/29,621	276/29,460	0.146	1.261	0.00 *
Dementia	492/29,749	895/29,118	0.126	0.261	0.00 *
Eating disorder	264/29,734	144/29,783	0.011 *	1.279	0.025 *
Self-harm	688/29,463	574/29,302	0.251	0.811	0.487

* *p* < 0.05.

**Table 4:** Propensity score matching for cardiac transplant versus heart failure patients.

Characteristic	Before Matching			After Matching		
	Heart Failure Patients	Transplant Patients	Standard Difference	Heart Failure Patients	Transplant Patients	Standard Difference
Age at index, years	68.9 +/− 16.7	44.8 +/− 20.9	1.274	62.9 +/− 17.3	44.8 +/− 20.9	0.944
Male	164,631 (57.7%)	3344 (68.7%)	0.229	3360 (69.2%)	3333 (68.7%)	0.012
Race						
White	149,310 (52.4%)	3081 (63.3%)	0.223	3139 (64.7%)	3071 (63.3%)	0.029
American Indian or Alaska Native	507 (0.2%)	10 (.2%)	0.006	11 (.2%)	10 (0.2%)	0.004
Pacific Islander	1281 (0.4%)	10 (0.2%)	0.043	10 (0.2%)	10 (0.2%)	<0.001
Black or African American	24,349 (8.5%)	1054 (21.7%)	0.373	991 (20.4%)	1051 (21.6%)	0.03
Asian	7967 (2.8%)	84 (1.7%)	0.072	68 (1.4%)	84 (1.7%)	0.027
Essential hypertension	131,365 (46.1%)	2937 (60.3%)	0.289	2992 (61.6%)	2937 (60.5%)	0.023
Neoplasms	62,477 (21.9%)	1580 (32.5%)	0.239	1571 (32.4%)	1577 (32.5%)	0.003
Diabetes mellitus	73,369 (25.7%)	2106 (43.3%)	0.376	2099 (43.2%)	2098 (43.2%)	<0.001
Obesity and overweight	46,002 (16.1%)	1509 (31.0%)	0.356	1536 (31.6%)	1506 (31.0%)	0.013
Psychosocial stressors	13,542 (4.7%)	391 (8.0%)	0.135	348 (7.2%)	391 (8.1%)	0.033
Chronic lower respiratory diseases	60,902 (21.4%)	1339 (27.5%)	0.144	1333 (27.5%)	1338 (27.6%)	0.002
Epilepsy or seizures	6062 (2.1%)	208 (4.3%)	0.122	162 (3.3%)	206 (4.2%)	0.047
Cerebral infarction	20,026 (7.0%)	853 (17.5%)	0.324	774 (15.9%)	848 (17.5%)	0.041
Ischemic heart diseases	111,021 (38.9%)	3002 (61.7%)	0.467	3012 (62.0%)	2999 (61.8%)	0.006
Heart failure	97,555 (34.2%)	4512 (92.7%)	1.529	4483 (92.3%)	4500 (92.7%)	0.013
Atrial fibrillation and flutter	70,600 (24.8%)	2545 (52.3%)	0.59	2475 (51.0%)	2535 (52.2%)	0.025
Cardiomyopathy	42,890 (15.0%)	4089 (84.0%)	1.905	4115 (84.8%)	4077 (84.0%)	0.022
Fibrosis and cirrhosis of liver	5488 (1.9%)	383 (7.9%)	0.278	357 (7.4%)	372 (7.7%)	0.012
Chronic kidney disease and acute kidney failure	75,401 (26.4%)	3411 (70.1%)	0.971	3389 (69.8%)	3399 (70.0%)	0.004
Number of patients	285,200	4867		4855	4855	

**Table 5:** Incidence of MHDs/ SUDs in cardiac transplant versus heart failure patients.

	Incidence			Survival Analysis	
Disorder	Incidence in Post-Heart Transplant Patients	Incidence in Control (Heart Failure)	*p*-Value for Incidence	Hazard Ratio	Hazard *p*-Value
	Expressed as the Total Number in the Propensity-Matched Cohort	Expressed as the Total Number in the Propensity-Matched Cohort			
Somatoform disorder	66/4822	32/4810	0.425	1.644	0.845
Anxiety	270/4531	138/4658	0.391	1.603	0.059
Panic disorder	109/4656	63/4757	0.29	1.394	0.067
PTSD	116/4612	72/4781	0.346	1.307	0.737
Adjustment disorder	292/4108	180/4633	0.046	1.527	0.00 *
Depression	645/3416	468/3642	0 *	1.217	0.094
Mood disorder	148/4736	95/4772	0.261	3.191	0.00 *
Nicotine dependence	255/4233	258/3708	0.168	0.671	0.281
Alcohol use disorder	134/4456	184/4206	0.64	0.554	0.072
Opioid use	144/4769	67/4726	0.038 *	1.791	0.009 *
Dementia	48/4834	168/4693	0.993	0.215	0.878
Eating disorder	65/4782	42/4810	0.08	1.234	0.462
Self-harm	134/4707	174/4675	0.123	0.605	0.786

* *p* < 0.05.

**Table 6:** Propensity score matching for VAD versus heart failure patients.

Characteristic	Before Matching			After Matching		
	Heart Failure Patients	VAD Patients	Standard Difference	Heart Failure Patients	VAD Patients	Standard Difference
Age at index, years	34.1 +/− 20.3	61.3 +/− 16.4	1.469	38.1 +/− 22.2	58.2 +/− 17.7	1.004
Male	18,737 (34.6%)	17,882 (66.6%)	0.675	6700 (62.9%)	6435 (60.4%)	0.051
Race						
White	26,699 (50.3%)	17,195 (64.0%)	0.28	6320 (59.3%)	6565 (61.6%)	0.047
American Indian or Alaska Native	94 (0.2%)	121 (0.5%)	0.049	53 (0.5%)	48 (0.5%)	0.007
Pacific Islander	52 (0.1%)	63 (0.2%)	0.34	22 (0.2%)	22 (0.2%)	<0.001
Black or African American	9854 (18.6%)	5629 (21%)	0.06	2174 (20.4%)	2096 (19.6%)	0.018
Asian	2205 (4.2%)	430 (1.6%)	0.153	238 (2.2%)	225 (2.1%)	0.008
Essential hypertension	10,839 (20.4%)	14,295 (53.2%)	0.723	3522 (33.0%)	3163 (29.7%)	0.073
Neoplasms	8744 (16.5%)	7244 (27%)	0.257	1927 (18.1%)	1811 (17.0%)	0.029
Diabetes mellitus	5799 (10.9%)	9246 (34.4%)	0.585	1892 (17.8%)	1764 (16.5%)	0.032
Obesity and overweight	8238 (15.3%)	7305 (27.2%)	0.293	1515 (14.2%)	1536 (14.4%)	0.006
Psychosocial stressors	2631 (5.0%)	1983 (7.4%)	0.101	468 (4.4%)	449 (4.2%)	0.009
Chronic lower respiratory diseases	9088 (17.1%)	6932 (25.8%)	0.213	1615 (15.2%)	1595 (15.0%)	0.005
Epilepsy or seizures	1722 (3.2%)	803 (3.0%)	0.015	322 (3.0%)	275 (2.6%)	0.027
Cerebral infarction	1209 (2.3%)	3359 (12.5%)	0.399	717 (6.7%)	649 (6.1%)	0.026
Ischemic heart diseases	2482 (4.7%)	14,479 (53.9%)	1.286	2028 (19.0%)	1899 (17.8%)	0.031
Heart failure	1537 (2.9%)	15,385 (57.3%)	1.472	1460 (13.7%)	1551 (14.6%)	0.025
Atrial fibrillation and flutter	1005 (1.9%)	9932 (36.2%)	0.993	915 (8.6%)	1027 (9.6%)	0.037
Cardiomyopathy	774 (1.5%)	9733 (36.2%)	0.993	722 (6.8%)	869 (8.2%)	0.052
Fibrosis and cirrhosis of liver	606 (1.1%)	929 (3.5%)	0.155	219 (2.1%)	207 (1.9%)	0.008
Chronic kidney disease and acute kidney failure	2924 (5.5%)	10,741 (40.0%)	0.902	1609 (15.1%)	1477 (13.9%)	0.035
Number of patients	53,119	26,866		10,659	10,659	

**Table 7:** Incidence of MHDs/ SUDs in VAD versus heart failure patients.

	Incidence			Survival Analysis	
Disorder	Incidence in VAD Patients	Incidence in Control (Heart Failure)	*p*-Value for Incidence Analysis	Hazard Ratio	Hazard Ratio *p*-Value
	Expressed as the Total Number in the Propensity-Matched Cohort	Expressed as the Total Number in the Propensity-Matched Cohort			
Somatoform disorder	62/10,596	64/10,568	0.722	1.6	0.642
Anxiety	265/10,341	345/10,120	0.011 *	0.9	0.044 *
Panic disorder	117/10,453	127/10,453	0.014 *	1.1	0.013 *
PTSD	125/10,456	140/10,448	0.056	1.1	0.02 *
Adjustment disorder	429/10,209	226/10,360	0.087	2.3	0.00 *
Depression	1035 /8849	813/8750	0.003 *	1.6	0.00 *
Mood disorder	174/10,509	95/10,496	0.286	2.1	0.82
Nicotine dependence	452/8868	540/8226	0.212	0.9	0.00 *
Alcohol use disorder	300/10,004	239/9818	0.873	1.4	0.00 *
Opioid use	134/10,466	206/10,258	0.563	0.74	0.001 *
Dementia	166/10,500	71/10,584	0.598	2.75	0.138
Eating disorder	63/10,592	43/10,569	0.779	1.62	0.031 *
Self-harm	207/10,404	145/10,405	0.057	1.7	0.03 *

* *p* < 0.05.

## Data Availability

Data will be made available upon request.
